# Impact of solutions and storage time on the chemical and mechanical properties of human dentin

**DOI:** 10.4317/jced.62433

**Published:** 2025-04-01

**Authors:** Fernanda Castelo Branco Santos Bettero, Camila de Carvalho Almança Lopes, Gabriel Júlio Guerra, Veridiana Resende Novais

**Affiliations:** 1 Postgraduation Program in Dentistry, Faculty of Dentistry, Federal University of Uberlândia, Uberlândia, MG, Brazil; 2 UNA School of Dentistry, Uberlândia, MG, Brazil; 3 Faculty of Dentistry, Federal University of Uberlândia, Uberlândia, MG, Brazil; 4 Department of Operative Dentistry and Dental Materials, School of Dentistry, Federal University of Uberlândia, Uberlândia, MG, Brazil

## Abstract

**Background:**

Considering the importance of standardization of the pH of control solutions and its impact on the chemical composition and mechanical properties of dentin during storage over a specified period, this study aimed to analyze the pH of control solutions and how it affects the chemical composition and mechanical properties of dentin stored over a given period.

**Material and Methods:**

Six control solutions—coconut water, mineral water, distilled water, deionized water, artificial tears, and saline—were kept in a bacteriological incubator at 37°C, with their pH measured using a pH meter (mPA-210 from MS Tecnopon ®) for seven days: T0 (initial), T1 (2 hours), T2 (24 hours), T3 (48 hours), and T4 (7 days). In the second phase, the two solutions with the most stable pH in the first phase were selected and aligned with the critical pH of dentin (pH ≥ 6.5). Human third molars were sectioned and divided into two groups (n=13): distilled water and deionized water. The pH of the solutions, the chemical composition, and the microhardness of the dentin were evaluated by pH meter, Fourier transform infrared spectroscopy (FTIR), and Knoop microhardness (KH), respectively, at the aforementioned time points. The values were analyzed by two-way ANOVA (storage time and solution), followed by the Tukey test.

**Results:**

Both solutions presented pH incompatible with the dentin until T2, and the samples presented a 40% reduction in microhardness at T4. Additionally, a reduction in carbonate and an increase in amides were observed in the dentin, indicating changes in the mineral and organic phases.

**Conclusions:**

It is concluded that both the solutions and the storage time negatively affect the chemical composition and microhardness of the dentin, highlighting the importance of carefully selecting the control solutions and the storage time in in vitro studies.

** Key words:**Dentin, hardness, FTIR, pH, storage solution.

## Introduction

A suiTable storage solution is essential in *in vitro* scientific research that evaluates dental structure, as it guarantees reliable results and accurate interpretation of the data. It is crucial as a baseline reference, allowing comparison between the effects of different treatments on tooth structure without generating changes in it (1). With a control group in a standard solution, the specific effects of the treatments under study can be evaluated, minimizing the confounding variables and ensuring that observed differences are only due to the tested treatments (2).

Dentin is composed of fluid (10%), collagen (20%), and hydroxyapatite (70%) (3). The pH of the medium in which it is immersed may depend on its composition and properties (4). During storage, ionic exchanges may occur between the medium and hydroxyapatite, especially at low pH, leading to the acidic dissolution of the crystals (5). Therefore, the chemical composition, pH, and storage time determine whether the solution can be used as a control in studies *in vitro* with human dentin. The optimum pH for dentin is below 6.5, fluctuating according to the exposure length so that the storage solution stays sTable during the research without interfering with the chemical composition of the tooth (6).

Control solutions and storage time are not standardized for preserving dental characteristics. Several solutions are listed in the literature, such as coconut water (7), deionized water (8), distilled water (9), mineral water (10), saline solution (11), and artificial tears (11). Therefore, this study aims to analyze the pH of different control solutions used in scientific research, investigating the influence on their chemical composition by FTIR and on the mechanical properties by Knoop microhardness (KH) of dentin over storage time. The null hypotheses are: (1) the control solutions did not alter the mechanical properties and chemical composition of dentin; (2) storage time does not influence the mechanical properties and chemical composition of dentin.

## Material and Methods

-First Phase

pH measurement

**Figure 1:**
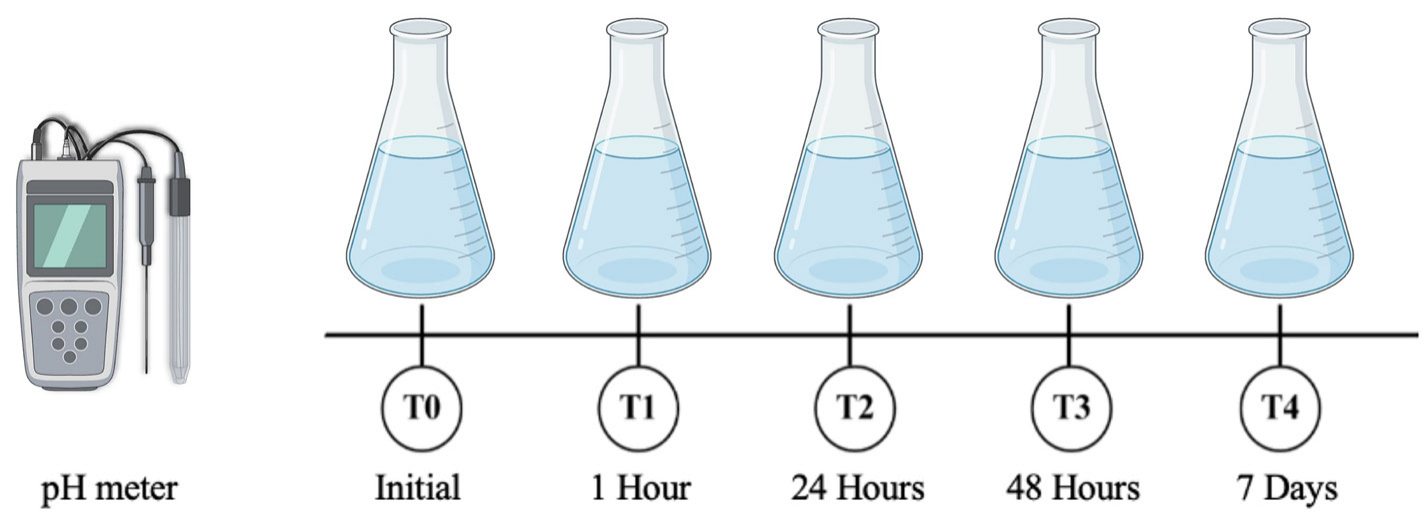
Schematic illustration of pH measurement of tested solutions.

pH measurement was performed with a pH meter (MS Tecnopon® mPA-210) in the following storage solutions: coconut water (Assiflora®), deionized and distilled water (collected at the Center for Research in Biomechanics, Biomaterials and Dental Cell Biology of the Federal University of Uberlândia), mineral water (Evian®), saline solution (Sorimax®) and artificial tear (Lacrifilm®). The pH meter was calibrated at the beginning of each test with pH 7.0 and pH 4.0 buffer solution (Quimica Contemporânea Dinâmica®). The pH measurements were performed at the following time intervals: T0 (initial), T1 (2 hours), T2 (24 hours), T3 (48 hours) and T4 (7 days). Throughout the experiment, the solutions were kept in a bacteriological incubator (Stufa Bacteriológica SL 101 - SOLAB) at 37ºC. This approach ensured the accuracy and consistency of the pH depth throughout the study (Fig. 1).

-Second Phase

Sample preparation

**Figure 2:**
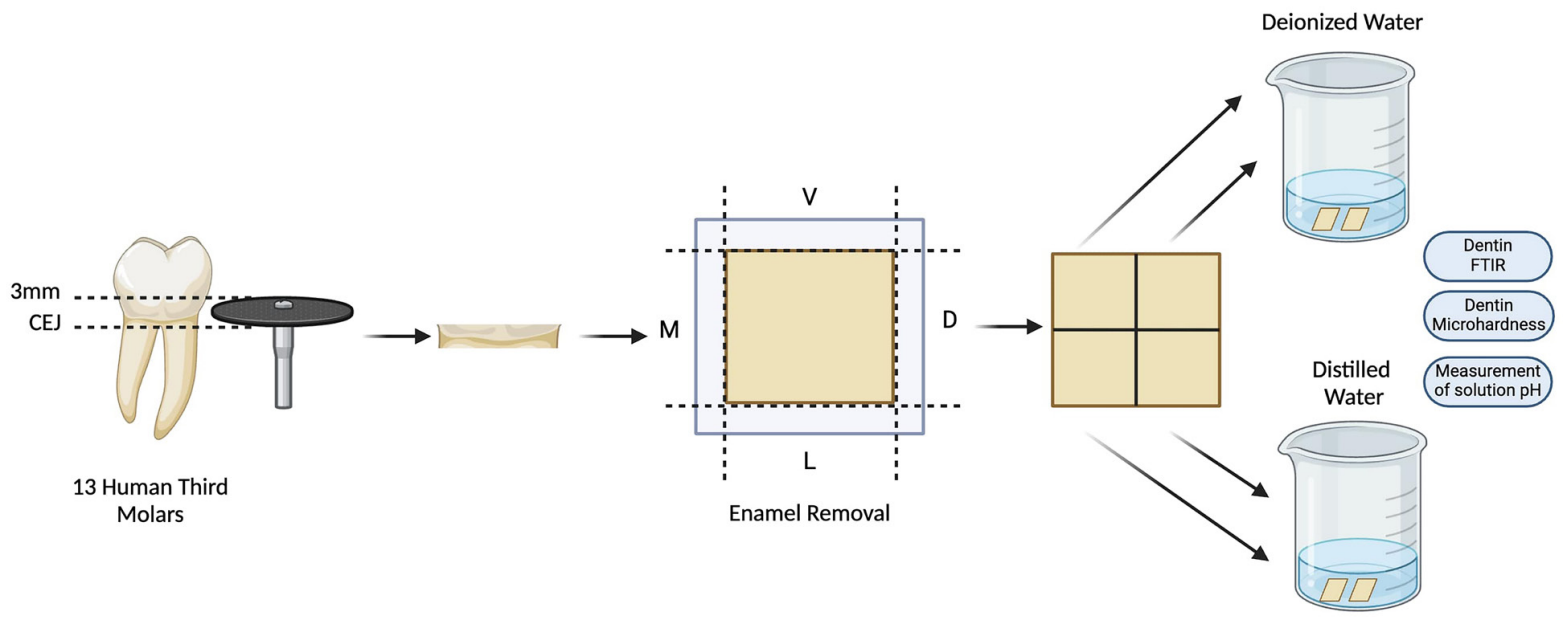
Schematic tensile illustration of sample preparation.

Three non-carious human third molars were collected, cleaned, and stored in distilled water at 4°C for at least 3 months after removal (12). This study was approved by the Research Ethics Committee of the Federal University of Uberlândia (Protocol CAAE 43234221.4.0000.5152) and informed consent was obtained from patients. In sample preparation, the teeth were sectioned with a water-cooled diamond disk (Isomet, 15HC diamond; Buehler Ltd., Lake Bluff, IL, USA) mounted on a precision cutter (Isomet 1000, Buehler Ltd., Lake Bluff, IL, USA). Two perpendicular cuts were made along the axis of each tooth at the specific cementoenamel junction, separating the crown from the root and 3mm toward the crown (Fig. 2). The enamel was removed, obtaining only dentin specimens. All slices were cut longitudinally in the mesial-distal and bucco-palatine axis, resulting in four specimens. Two were stored in deionized water and two in distilled water. The pH of the solutions was monitored at indicated time intervals.

Storage medium and sample size determination

The samples were divided into two groups, considering the following storage solutions: deionized water and distilled water, selected for presenting stable pH and aligned with the critical pH of dentin, as discovered in the initial phase of this study. The samples were stored in 5 ml of each solution, enough for complete submersion throughout the experiment, and kept in a bacteriological incubator (Stufa Bacteriológica SL 101 - SOLAB brand) at 37º C. The evaluation of the pH of the solution, chemical composition (FTIR), and dentin microhardness (KH) occurred at the following times: T0 (initial), T1 (2 hours), T2 (24 hours), T3 (48 hours) and T4 (7 days).

Microhardness test

To evaluate the Knoop microhardness (KH) of dentin, the samples were embedded in polystyrene resin (AM 190 resins; Aerojet, São Paulo, SP, Brazil). The surfaces were sanded with silicon carbide paper in 600, 800, 1200, and 2000 grits (Norton, Campinas, SP, Brazil) and polished with felt discs and metallographic diamond pastes (grits 6, 3, 1, and ¼ μm; Arotec, São Paulo, SP, Brazil) (12). The entire process was timed and performed by a single operator. Knoop microhardness values were determined with a microhardness tester (FM 700; FutureTech Corp., Kawasaki, Japan) (13) by applying a load of 100g for 10s (14). Three indentations were made on each specimen, with 100μm between them, and the projections were averaged to determine the KH of the specimens.

ATR/FTIR

The chemical composition of the samples was determined using ATR/FTIR (Vertex 70, Bruker, Ettlingen, Germany). The dentin surfaces were positioned against the diamond crystal of the ATR/FTIR unit and pressed with a force gauge at a constant pressure to ensure contact. Spectra were recorded from 400 to 4,000 cm-1 with a resolution of 4 cm-1. Each sample was tested 32 times, and the final spectrum was the average of these analyses. The spectra were recorded and analyzed by OPUS 6.5 software (Bruker, Ettlingen, DEU). After baseline correction and normalization, the area under each band was integrated using the software tools. The bands evaluated were amide I (C=O at 1655 cm-1 ), amide II ( NH bending and CN stretching at 1544 cm-1 ), amide III ( CN at 1235 cm-1 ), phosphate V 1 and V 3 ( 960 cm-1 and 1040 cm-1 ) and carbonate V2 ( 872cm-1 ). The following parameters were evaluated: (1) phosphate/amide ratio I (ratio of the integrated areas at 1035 and 1655 cm-1, attributed to the V1 vibration and V3 of the phosphate ion and the C=O stretching of collagen amide I, respectively); (2) phosphate/carbonate ratio (ratio of the integrated areas of the phosphate V1 and V3 at 1,035 cm-1 and carbonate v2 at 872 cm-1) (14).

-Statistical Analysis

The data were tested for normal distribution (Shapiro-Wilk test, *p* > 0.05) and equality of variances (Levene’s test, *p* > 0.05), passing both tests. The pH, ATR/FTIR data, and KH were analyzed by variance analysis (two-way ANOVA with repeated measures), considering the factors time (T1, T2, T3, and T4) and storage solution (deionized water and distilled water), applied Tukey’s post hoc test. The significance level was 5%. All statistical analyses were performed using the SigmaPlot software (version 12.0, Systat Software, Inc., San Jose, CA, USA).

## Results

-First Phase Results

Coconut water exhibited a gradual decrease in pH from 5.10 at T0 to 4.60 at T3, followed by a slight increase to 4.90 at T4. Deionized and distilled water maintained relatively sTable pH levels, with a slight increase over time, reaching 6.68 and 7.06, respectively, at T4. Mineral water consistently displayed the highest pH, increasing slightly from 7.61 at T0 to 7.81 at T4. Saline solution showed a notable dip in pH at T2 (5.47) but recovered to its initial value (6.02) by T4. Artificial tear solution remained stable overall, with minor fluctuations and ending at 6.80 at T4. These results demonstrate the dynamic pH changes of the solutions over time, likely influenced by their compositions and environmental factors ( Table 1).

-Second Phase Results

pH measurements

The mean and standard deviations of the pH values obtained are presented in  Table 2. Two-way ANOVA revealed a significant difference for the storage solution (*p*=0.02), for time (*p*<0.001), and for the interaction between solution and time (*p*=0.002). After storage, it was observed that deionized water and distilled water obtained similar pH values at T1. An increase in pH was observed in both solutions from T2 onwards, with higher values for distilled water. Deionized water presented T3 values similar to T2 and T4, with T4 values higher than T2 values. Distilled water exhibited a significant increase in pH from T2, T3, and T4, presenting the highest pH values. In both solutions, a progressive increase in pH values was observed throughout the analysis period.

Knoop Microhardness - KH

The standard deviation of the KH considering the solution and storage time is detailed in  Table 3. The two-way analysis of variance indicated significant differences for storage time (*p* < 0.001) and did not show statistical significance between the means of the storage solutions (*p*= 0.027) and the interaction between the factors for KH (*p*= 0.650). Dentin stored in deionized water presented similar hardness values compared to distilled water. KH values were obtained for both solutions in all time frames evaluated up to T3. However, the values decreased significantly from T4 onwards, differing from the other storage periods.

ATR/FTIR

The average values and standard deviations for vibration modes and composition or chemical conFigurations found by ATR /FTIR are presented in  Table 4. For amides I, II, and III, two-way ANOVA revealed significant differences for storage time (*p* = 0.002; *p* = < 0.001; *p*=0.010) but not for storage solution (*p* = 0.096; *p* = 0.644; *p* = 0.338) nor the interaction between the factors developed (*p* = 0.067; *p* = 0.287; 0.275). Amide values increased from T2 onwards, with T2 being similar to T1, which presented the lowest values, and T3 and T4, which reached the highest values for both solutions. Amide II and III increased from T2, with T2 similar to T3 and T4. The values of amides I, II, and III reached the highest after 48 hours (T3) from the beginning of the analysis. For phosphate levels, two-way ANOVA revealed no significant differences for storage time, storage solution, and the interaction between the factors analyzed (*p* = 0.106; *p*= 0.279; *p*=0.308). Carbonate showed a significant difference for storage time (*p*<0.001) and for storage solution (*p*=0.007), but there was no significant difference in the interaction between these factors (*p*=0.816). A decrease in carbonate was observed in both solutions from T2, with higher values for distilled water. For both solutions, T2 values were similar to T1, with lower values, and T3 and T4, with higher values.

The M/M ratio did not reveal significant differences in storage time, storage solution, and interaction between analytical factors (*p*=0.263; *p*=0.718; *p*=0.259). In the M/C ratio, a significant difference was observed for storage time (*p* < 0.001) and storage solution (*p* < 0.001), with no statistical difference in the interaction between them (*p*=0.769) ( Table 4). An increase in the phosphate/carbonate ratio was observed in both solutions from T2 onwards, with higher values for distilled water ( Table 4).

## Discussion

The tested null hypotheses in this study were rejected, showing that the control solutions impacted the mechanical properties and chemical composition of dentin. Furthermore, it was found that the storage time influenced not only the microhardness but also the chemical characteristics of dentin.

In the first stage, after reviewing the literature on the primary control solutions used in scientific research, six solutions were selected, and their pH was measured over seven days. This approach allowed us to understand the variations and stability of pH over time. Distilled and deionized water presented a pH compatible with dentin over the seven days, leading to the selection of these solutions for a more comprehensive analysis. Distilled water is obtained through evaporation followed by condensation, which purifies water, removing organic and inorganic components without purifying it exclusively into H2O, such as ultrapure water (15). The distillation process gives the water a lower buffering capacity by removing bicarbonate and phosphate ions, which are essential for this function (16).

On the other hand, deionized water results from removing all dissolved minerals and impurities, such as chloride, calcium, sodium, and magnesium. However, some organic components may remain since deionization mainly removes ions (15). However, deionization can leave traces of ions, contributing to this solution’s buffering capacity (16).

Hydroxyapatite (HA), the main mineral component of dentin, can undergo ion substitutions such as Ca2+ for Na+, PO43- for CO32-, and OH- for CO32-, F- and Cl-, depending on storage conditions (17). Calcium phosphate hydroxyapatite is considered a model for biological mineralization, although its ideal formula has not yet been conclusively identified (18). Studies show that the composition of crystalline apatites varies due to the substitution of anions and cations and different types of ionic vacancies. Understanding these complexities is crucial to understanding the mechanical properties and *in vivo* behavior of hydroxyapatite (18).

**Figure 3:**
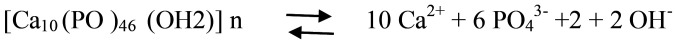
Formula.

At a pH equal to or less than 6.5, dentin can undergo demineralization, resulting in the loss of minerals such as calcium and phosphate (4). At baseline, both solutions had a lower pH compatible with dentin (deionized water with pH 6.05 and distilled water with pH 6.22). The increase in pH after storage occurred due to the release of tooth minerals into the water, especially distilled water, as it has a lower buffering capacity. The dissolution of hydroxyapatite, especially carbonates, releases ionic species that, when in contact with water, neutralize hydrogen ions (H+), making the pH more alkaline (19,20) Considering that hydroxyapatite is the tooth mineral, its dissolution in aqueous medium can be exemplified by the equation (Aoba, 2017): (Fig. 3).

The presented equation shows the dissolution of the mineral alone (on the left) into soluble ions (on the right) in solution. According to Le Chatelier’s principle, if the concentration is supersaturated compared to water, the mineral will dissolve until it reaches the equilibrium described in the solution (19).

The study observed significant changes in the microhardness of dentin exposed to deionized and distilled water, corroborating the results of the pH assessment. The micromechanical properties of dentin, assessed by a dynamic microhardness indenter, indirectly reflect the amount of minerals in the tooth structure (21). In both solutions, the initial pH below 6.5 suggests a demineralizing effect. Notably, deionized and distilled water, because they do not contain calcium and phosphate, exhibited a high capacity to dissolve the mineralized phase of dentin, being considered the leading cause of demineralization and tissue softening (8). The samples showed a significant decrease in microhardness after seven days of storage, with a 40% reduction for both solutions. Another study that used deionized water as a storage medium found changes in stored dentin, with decreased mechanical properties after one day and a drop below 50% after one week, attributed to demineralization during storage (8).

The chemical composition of the tooth was verified by Fourier Transform Infrared Spectroscopy (ATR/FTIR) to identify possible mineral changes in the dentin after storage in control solutions. Sometimes, ratios between the reference areas result in comparison values that exclude the uncertainties of the sample variation (22). With the ATR/FTIR data, it was possible to calculate the phosphate/amide I (M/M) ratio indexes, indicating the relationship between the inorganic and organic phases. In contrast, the phosphate/carbonate (M/C) ratio represents the relationship between the inorganic components, providing additional information about the structure of hydroxyapatite. This study observed changes in the composition or chemistry exposed to deionized and distilled water, corroborating the pH and KH results.

The organic phase of dentin, predominantly composed of type I collagen, presents spectral bands of amides I, II, and III (23). The results of this investigation indicate that the storage time influenced the amide values, with no difference between the storage solutions. The increase in amide levels detected by ATR/FTIR can be explained by the analysis of the specific properties of the amide and its molecular structure, as well as by the comparison with the results obtained in the hardness depths. According to the KH results, dentin demineralization occurred, causing a drop in hardness values during the analysis. Demineralization exposes other dentin compounds, such as amide, justifying the increase in amide identified by ATR/FTIR in the chemical composition of dentin (23). Dentin collagen establishes covalent and intermolecular cross-bonds to maintain its sTable network structure (24). The edges of these bonds can interact with free radicals, increasing amide formation (25). Since amides are essential compounds (26), their detection by Fourier transform infrared spectroscopy becomes more prominent during solution storage and dentin demineralization. It can be inferred, therefore, that alkalinization of the medium may also be a consequence of the exposure of amide to the environment (19).

In the initial stage of the study, emphasis was placed on evaluating the pH stability of the most used solutions for dentin storage in *in vitro* research. This procedure aims to identify the solutions with the most sTable pH and to use them in subsequent analysis on the interaction and changes in the mineral component of dentin in contact with the storage medium. When a dentin sample was stored in the solutions, an imbalance occurred due to the difference in the concentration of the substance (19). In addition, both solutions maintained a pH incompatible with dentin (pH < 6.5) for up to two hours of storage, contributing to decreased carbonate. Demineralization is a chemical phenomenon in which mineral ions, such as phosphate and carbonate, present in the tooth structure, are distributed in the water, moving from the most concentrated to the least concentrated medium (7,8). Carbonate ions (CO²) are less present within the hydroxyapatite crystal matrix when compared to phosphate ions (PO³). Incorporating carbonate into the hydroxyapatite structure creates a crystal matrix more susceptible to dissolution in acidic environments. In acidic conditions, carbonate ions react quickly with hydrogen ions (H+), forming carbonic acid (HCO), which dissociates into water and carbon dioxide (CO). This process reduces the presence of carbonate in the mineral structure, promoting its preferential loss compared to phosphate, which increases the solubility of hydroxyapatite in an acidic medium (27). This was observed in this study since there was no statistically significant difference in phosphate values over time and between the different solutions evaluated, decreasing the greater stability of phosphate ions in the hydroxyapatite structure compared to carbonate ions. In addition, there was an increase in carbonate levels during the studied period, with reduced values for distilled water.

Calculating the phosphate/amide ratio I by ATR/FTIR is essential for assessing the structural integrity and chemical composition of dentin, as it offers a relative measure between the inorganic (phosphate) and organic (collagen) components. This ratio is widely used in studies that analyze the mineralization state of dentin since variations may indicate demineralization (28). In the present study, no statistically significant differences were found in the phosphate/amide ratio over time or between the solutions tested, indicating stability in the mineral structure of dentin under the conditions evaluated.

Furthermore, the phosphate/carbonate ratio is critical as it provides an understanding of the proportion of carbonate relative to phosphate and may indicate changes in the mineralization process, which can affect dentin’s mechanical strength (29).

In this study, significant differences were observed between the solutions, with distilled water presenting higher values in the phosphate/carbonate ratio compared to deionized water. This suggests a greater susceptibility of the dentin mineral structure to carbonate release when stored in distilled water. However, there were no significant differences over the storage time, which indicates that the composition of the solutions had a more expressive impact on the carbonate dynamics than the storage duration.

It has been shown that control solutions, before being considered occasional, can cause substantial changes in dentin structure. This fact highlights the importance of methodological rigor and continuous evaluation of these periods to ensure reliable and significant results in dental research. The choice of storage solution is crucial in *in vitro* investigation of tooth structure, specifically as a reference for comparing the effects of tested variations or treatments, ensuring uniform conditions (30). For future research, it is recommended that a control solution with criteria, strict pH control, detailed temporal monitoring, and inclusion of additional parameters be selected, aiming to mitigate the observed effects and contribute to more reliable and applicable advances in dental research.

## Conclusions

This *in vitro* study indicated a progressive increase in pH in both solutions (distilled and deionized water) over time, decreasing a tendency towards alkalinization of the medium. Dentin stored in deionized water presented hardness values similar to those observed in distilled water, revealing that both impacted microhardness in a formative way. Knoop hardness (KH) values remained unchanged for up to four and eight hours (T3) with a significant reduction in one week (T4), which suggests a progressive distribution of mechanical properties over prolonged storage times.

Chemical analysis by ATR/FTIR revealed significant changes in the chemical composition of dentin, especially in the levels of amides and carbonates, highlighting the importance of collagen for the structural structure of dentin. The alkalinization of the medium, associated with the exposure of amides, offers an additional understanding of the observed chemical changes.

Therefore, future investigations should emphasize carefully selecting control solutions and monitoring experimental periods, ensuring consistent and applicable advances in dental research.

## Figures and Tables

**Table 1:** pH of the solutions supplied during the investigated time.

SAMPLE/T	T0	T1	T2	T3	T4
Coconut Water	5.10	5.00	4.73	4.60	4.90
Deionized Water	6.10	6.06	6.60	6.65	6.68
Distilled water	6.19	6.66	6.61	6.75	7.06
Mineral Water	7.61	7.70	7.42	7.79	7.81
Saline solution	6.02	6.00	5.47	5.90	6.02
Artificial Tear	6.87	6.82	6.70	6.56	6.80

**Table 2:** Mean and standard deviation of pH values.

SOLUTIONS/TIME	T0	T1	T2	T3	T4
Deionized Water	6.05 (0.07)	6.08 (0.10) Ac	6.40 (0.13) Bb	6.49 (0.19) Bab	6.52 (0.16) Ba
Distilled water	6.22 (0.19)	6.00 (0.15) Ac	6.50 (0.19) Ab	6.63 (0.22) Aa	6.67 (0.19) Aa

*Different capital letters ( analysis in columns) and lowercase letters ( analysis in rows) represent significant differences (*p*>0.05).

**Table 3:** Mean and standard deviation of microhardness values.

SOLUTIONS/TIME	T0	T1	T2	T3	T4
Deionized Water	48.08 (7.62)	47.29 (9.10) Aa	47.61 (6.22) Aa	48.51 (6.50) Aa	28.53 (4.71) Ab
Distilled water	50.46 (6.69)	50.31 (6.21) Aa	49.89 (7.28) Aa	50.25 (6.10) Aa	30.61 (5.76) Ab

*Different capital letters (analysis in columns) and lowercase letters (analysis in rows) represent significant differences (*p*>0.05).

**Table 4:** Means and standard deviations of the integrated area of each chemical component, M/M ratio, and phosphate/carbonate ratio analyzed by ATR/FTIR.

Amide I	T0	T1	T2	T3	T4
Deionized Water	2.88 (0.43)	3.25 (0.61) Ab	3.25 (0.49) Aab	3.63 (0.56) Aa	3.62 (0.46) Aa
Distilled water	2.63 (0.32)	2.82 (0.42) Ab	3.29 (0.57) Aab	3.45 (0.68) Aa	3.35 (0.58) Aa
Amide II	T0	T1	T2	T3	T4
Deionized Water	0.36 (0.14)	0.58 (0.24) Ab	0.67 (0.26) Aa	0.77 (0.32) Aa	0.69 (0.30) Aa
Distilled water	0.27 (0.11)	0.51 (0.11) Ab	0.69 (0.17) Aa	0.69 (0.20) Aa	0.67 (0.15) Aa
Amide III	T0	T1	T2	T3	T4
Deionized Water	0.37 (0.15)	0.51 (0.26) Ab	0.67 (0.26) Aa	0.77 (0.32) Aa	0.69 (0.30) Aa
Distilled water	0.40 (0.18)	0.38 (0.11) Ab	0.53 (0.19) Aa	0.53 (0.15) Aa	0.54 (0.16) Aa
Phosphate	T0	T1	T2	T3	T4
Deionized Water	22.66 (0.37)	22.34 (0.56) Aa	22.37 (0.58) Aa	22.01 (0.66) Aa	21.92 (0.65) Aa
Distilled water	22.33 (0.38)	22.43 (0.64) Aa	22.33 (0.36) Aa	22.38 (0.32) Aa	22.36 (0.69) Aa
Carbonate	T0	T1	T2	T3	T4
Deionized Water	2.69 (0.33)	2.83 (0.42) Aa	2.68 (0.25) Aab	2.70 (0.21) Ab	2.44 (0.38) Ab
Distilled water	2.63 (0.23)	2.70 (0.26) Ba	2.53 (0.13) Bab	2,45 (0,13) Bb	2.43 (0.33) Bb
Phosphate/Amide I	T0	T1	T2	T3	T4
Deionized Water	8.01 (1.00)	7.04 (1.20) Aa	7.02 (1.00) Aa	6.21 (1.09) Aa	6.69 (1.06) Aa
Distilled water	8.60 (1.01)	8.13 (1.31) Aa	7.00 (1.37) Aa	6.88 (1.25) Aa	7.28 (1.45) Aa
Phosphate/Carbonate	T0	T1	T2	T3	T4
Deionized Water	8.51 (0.88)	8.00 (0.41) Bb	8.42 (0.90) Ba	8.19 (0.57) Ba	8.75 (0.60) Ba
Distilled water	8.59 (0.69)	8.38 (0.73) Ab	8.84 (0.43) Aa	8.93 (0.92) Aa	9.46 (0.71) Aa

*Different capital letters ( analysis in columns) and lowercase letters ( analysis in rows) represent significant differences (*p*>0.05).

## Data Availability

The datasets used and/or analyzed during the current study are available from the corresponding author.
